# What Influences Parental Engagement in Early Intervention? Parent, Program and Community Predictors of Enrolment, Retention and Involvement

**DOI:** 10.1007/s11121-018-0897-2

**Published:** 2018-04-09

**Authors:** Naomi J. Hackworth, Jan Matthews, Elizabeth M. Westrupp, Cattram Nguyen, Tracey Phan, Amanda Scicluna, Warren Cann, Donna Bethelsen, Shannon K. Bennetts, Jan M. Nicholson

**Affiliations:** 1 Parenting Research Centre, Level 5, 232 Victoria Parade, East Melbourne, VIC 3003 Australia; 2 Judith Lumley Centre, La Trobe University, Melbourne, VIC Australia; 3 Murdoch Children’s Research Institute, Parkville, VIC Australia; 4 Department of Paediatrics, The University of Melbourne, Parkville, VIC Australia; 5 School of Early Childhood, Queensland University of Technology, Kelvin Grove, Brisbane, QLD Australia

**Keywords:** Parent engagement, Parenting programs, Enrolment, Retention, Involvement

## Abstract

Poor participant engagement undermines individual and public health benefits of early intervention programs. This study assessed the extent to which three types of engagement (participant enrolment, retention and involvement) were influenced by individual, program and contextual factors. Data were from a cluster randomised controlled trial (*N* = 1447) of a community-based parenting program, delivered at two levels of intensity (group sessions with and without individualised home coaching) conducted in Victoria, Australia. Individual (parent and family) factors and program factors were assessed by parent report and administrative records, and contextual factors by area-level population statistics. Data were analysed using multilevel logistic or linear regression models. Individual and contextual factors predicted enrolment, while family and program factors were more influential on program retention and parents’ active involvement. Provision of individualised support was important to all forms of engagement, particularly for families experiencing the greatest barriers to participation. These findings indicate that different strategies are required to effectively support families in the processes of enrolling, continuing to attend and actively participating in early intervention programs.

**Electronic supplementary material:**

The online version of this article (10.1007/s11121-018-0897-2) contains supplementary material, which is available to authorized users.

Governments in developed countries are investing in early intervention and prevention programs to enhance the life chances of developmentally vulnerable children. Paradoxically, family risk factors that contribute to children’s vulnerability (e.g. socio-economic disadvantage, very young or single parenting, low parent education, minority background, parent mental health problems) are also associated with increased likelihood that parents will not enrol, actively participate or remain in prevention programs for their full duration (Axford et al. 2012; Miller and Prinz 2003; Morawska and Sanders 2006a). This presents a major challenge — effective programs will have limited individual and public health benefits and cost-effectiveness if they fail to engage the target population (Spoth et al. 2000). Previous studies of parental engagement with prevention programs have focused on recruitment, enrolment and attendance, with less attention on parents’ active engagement with the program content. Additionally, few studies have gone beyond assessing the individual socio-demographic determinants of engagement to include program and community factors (Axford et al. 2012; Whittaker and Cowley 2010). The current study addresses these limitations using data from the active intervention arms of a large randomised controlled trial of a community-based parenting program for disadvantaged families in Victoria, Australia (Nicholson et al. 2016). Specifically, this paper explores the individual, program and community factors associated with three aspects of parental engagement: enrolment, retention and involvement in program activities.

Children from socially and economically disadvantaged families have elevated risk for poor developmental outcomes (Bradley and Corwyn 2002; Victorino and Gauthier 2009), particularly in the areas of language, communication, literacy and longer term academic outcomes (Hart and Risley 1995; Nicholson et al. 2010). While a quality home learning environment protects against these risks (Landry et al. 2008), parents’ capacity to engage in development-enhancing interactions with their children may be compromised by the multiple challenges posed by socio-economic disadvantage (Garvey et al. 2006).

Parenting programs that aim to mitigate the developmental risks associated with socio-economic disadvantage have had only modest effects, attributed in part to poor uptake and high attrition (see Axford et al. 2012). Up to two thirds of families offered parenting programs do not enrol (Baker et al. 2011; Garvey et al. 2006), and a further 40–60% stop attending part way through the program (Axford et al. 2012; Morawska and Sanders 2006a). This may reflect the preventative focus with parents failing to see the need for assistance with a problem that does not currently exist, and to prioritise this over other competing demands (Axford et al. 2012; Garvey et al. 2006). A greater understanding of the factors that enhance or impede engagement is crucial to the effective provision of early intervention and prevention, particularly for low-income families.

## An Integrated Model of Participant Engagement

Research examining the patterns and determinants of parental engagement in prevention programs has been hampered by inconsistent terminology. Terms such as ‘recruitment’, ‘enrolment’, ‘attendance’, ‘retention’ and ‘completion’ are inconsistently defined and used somewhat interchangeably (Baker et al. 2011; Whittaker and Cowley 2010). For this paper, we build on the integrated conceptual model of participant engagement proposed by Matthews et al. (2011) to identify four distinct types of engagement. *Recruitment* may be defined as a population-level measure that considers the reach of the program to the target population, assessed as the proportion of the eligible target population who are approached and indicate an intention to attend. *Enrolment* refers to the proportion of those recruited who actually participate by turning up at least once; *retention* is the extent to which participants continue to attend across the duration of the program, and *involvement* is the extent to which participants actively practice and apply what they have learnt from the program. In the absence of data regarding program reach, the current paper focuses on the three latter measures of engagement: enrolment, retention and involvement.

## Individual Factors Impacting on Participant Engagement

A range of parent and family socio-demographic characteristics are associated with participant engagement (for reviews, see Morawska and Sanders 2006b; Whittaker and Cowley 2010). For example, fathers are much less likely to attend parenting programs than mothers (Cortis et al. 2009). Parents from dual earner families, low-income families and those with low education or from culturally and linguistically diverse backgrounds are less likely to be recruited into and attend parenting programs (Brown et al. 2012; Eisner and Meidert 2011; Heinrichs et al. 2005; Mendez et al. 2009; Sanders and Kirby 2012) and less likely to utilise program techniques afterwards (Eisner and Meidert 2011). Data on single parent families are mixed, with some studies finding that single parents are no less likely than parents from couple families to enrol and remain in parenting programs (e.g. Eisner and Meidert 2011; Heinrichs et al. 2005), while others report an under-representation of single parents (e.g. Gross et al. 2001).

Parent psychosocial factors also influence engagement. Positive attitudes and beliefs about help-seeking are associated with greater likelihood of ‘sign up’ (Cortis et al. 2009; Morawska and Sanders 2006b) as are positive previous experiences with services (Morawska and Sanders 2006b). Perceived benefits are associated with greater attendance and participation (McCurdy and Daro 2001). The association between child factors such as problematic behaviour or developmental concerns is less clear. In a review of parent engagement in behaviour management programs, Morawska and Sanders (2006a) reported that greater behaviour problems were associated with lower attendance and completion rates for some studies, while others reported increased participation with increasing problems. More recently, Eisner and Meidert (2011) found no association between perceived child behaviour problems and program sign up. In contrast, parental anxiety about being judged and feeling different or unwelcome due to class, race or cultural background are associated with decreased help-seeking and lower service uptake and continued attendance (Bussing et al. 2003; Cortis et al. 2009). While mental health would seem likely to affect engagement, three studies found no relationship between depressive symptoms and enrolment or retention (Baker et al. 2011; Garvey et al. 2006; Spoth et al. 1999).

## Program Factors Impacting on Participant Engagement

Program engagement research often describes engagement difficulties in terms of certain families being ‘*difficult to reach*’. Qualitative research indicates that for many, their experience is of a program that is ‘*difficult to access*’ (Cortis et al. 2009). Practical barriers such as work, childcare or transport difficulties can prevent attendance by parents who are interested and would like to participate in a program (Berthelsen et al. 2012; Mendez et al. 2009). While these may be regarded as parent characteristics, for this study, we conceptualise such barriers as ‘program factors’ in that they need to be managed in how the program is designed and delivered. In a review of mental health prevention programs, Ingoldsby (2010) identified 17 randomised controlled trials that systematically explored variations in program-level engagement strategies. Participation barriers were reduced by approaches that increased the bond between the service provider and potential participants (e.g. telephone or in-person pre-program contact) and multicomponent approaches that provided adjunct support (e.g. home visits, referral to additional services). Practitioner skills and cognitions have also been linked to increased program retention (Berthelsen et al. 2012; Cortis et al. 2009). In a review of 27 studies of group parenting programs, Whittaker and Cowley (2010) found that greater practitioner experience, skills in facilitation and understanding of program theory were associated with higher attendance and lower dropout. In contrast, low practitioner efficacy (Axford et al. 2012) and staff attrition (Cortis et al. 2009; Olds et al. 2015) were associated with low participant retention.

## Contextual Factors Impacting on Participant Engagement

Research exploring the effects of broader socio-environmental factors on engagement in parenting programs has been limited in scope and produced mixed findings. While individual levels of socio-economic disadvantage vary within communities, the evidence mostly indicates that neighbourhood socio-economic disadvantage is associated with lower reach and enrolment (see McCurdy and Daro 2001). Community-level resources that pose barriers to enrolment include poor public transport, large travel distances and social isolation (Berthelsen et al. 2012; Cortis et al. 2009), while greater social capital (i.e. strong community networks) enhances program enrolment (Eisner and Meidert 2011; McCurdy and Daro 2001).

## Measuring Participant Engagement

In engagement research, attendance is often used as a proxy measure for ‘dose’ or exposure to program content. This fails to capture important variations in participants’ active engagement with the program elements that are hypothesised to impact on outcomes (Brown et al. 2012; Whittaker and Cowley 2010). Even in programs structured to provide opportunities for practice, there can be considerable variation in individual participation in such activities. We refer to this type of engagement as ‘participant involvement’. Involvement has typically been measured as completion of homework tasks, or more recently, by assessing participants’ use of program content (Eisner and Meidert 2011).

In one of the few studies to explore determinants of multiple types of engagement, Eisner and Meidert (2011) found that predictors varied across enrolment, retention and involvement. Family characteristics (dual earner; more than two children) were associated with initial program enrolment, whereas group/program characteristics and parent-reported community connectedness predicted retention and involvement.

## The Current Study

The current study extends this work to investigate the impact of individual, program and community contextual factors on parent engagement with *smalltalk*, a community-based group program to assist disadvantaged parents to provide their children with an enriched home learning environment. The ultimate goal is the prevention of child difficulties in language, communication, literacy and school readiness (Hackworth et al. 2017). *smalltalk* was delivered through two existing early childhood service platforms: via Maternal and Child Health (MCH) parent education groups for parents of infants and via facilitated parent-child playgroups for parents of toddlers (see Nicholson et al. 2016). These universal services are the optimal platform for program delivery as they are available in all local government jurisdictions, and are well regarded and widely accessed by a diverse range of parents (see Nicholson et al. 2016 for more detail). *smalltalk* content was consistent across the two service platforms, but method of instruction and program duration varied to match the typical way that each platform provided parenting programs (see Table 1 for more detail). Briefly, for parents of infants, *smalltalk* was a stand-alone 6-week structured group parent education program; for parents of toddlers, *smalltalk* was a 10-week facilitated playgroup. For both, weekly sessions were 2 hours in duration. In each service, parents were recruited into one of two levels of intensity, according to their residential location: the group program alone (*smalltalk group-only*) or the group program accompanied by six home coaching sessions designed to strengthen the uptake and application of skills within the home (*smalltalk plus*).

**Table 1:** *smalltalk* program design and content

	Infant group program	Toddler group program	Home coaching (infant and toddler)
Child age range at commencement	6–12 months	12–36 months	6–12 months (infant); 12–36 months (toddler)
Delivery setting	Community venue	Community venue	Home
Format	Maternal & Child Health (MCH) parenting group	Supported playgroup (parent and child attended)	Individual
No of sessions offered	6	10	6
Duration of session	2 hours	2 hours	2 hours
Program content (5 domains)	Opportunities for child learning via everyday parent-child interactions, stimulating home environment, parent self-care, parenting confidence, connectedness to community and services		
Mode of delivery	Facilitated group discussion	Incidental learning opportunities in individual and small group interactions	Coaching DVD and filmed in-home practice
Facilitator	MCH nurse or MCH service worker	Community worker/playgroup facilitator	MCH nurse or community worker
Facilitator training	2-day group training plus individual post-training support	2-day group training plus individual post-training support	2-day group training plus individual post-training support

We made the following hypotheses. Firstly, as there was a strong focus throughout program development and staff training on tailoring to the needs of vulnerable families, we expected that parent/family indicators of disadvantage would not influence engagement for those parents who attended. We therefore hypothesised that parent/family factors would be related to poorer initial enrolment as found in previous studies, but would not be associated with poorer retention and involvement. Second, we hypothesised that fewer participation barriers, more favourable program factors and less community-level disadvantage would be associated with greater retention and involvement.

Individualised home coaching as an adjunct to the group program provided the opportunity for home coaches to encourage parental participation in the groups. However, this combined group plus home-based approach had not been used before by the services, and they expressed two concerns that this could reduce enrolment due to parents’ reluctance to have someone in their home, and that those who received home visits might not see a need to go to the group as well. We therefore explored whether home coaching had unintended adverse effects on enrolment, attendance and involvement.

## Method

### Study Design

Data were collected as part of a government-funded evaluation (Hackworth et al. 2017; Nicholson et al. 2016). Two parallel cluster RCTs were conducted evaluating *smalltalk* in the MCH service for parents of infants aged 6–12 months (infant RCT) and in the facilitated playgroup service for parents of toddlers aged 12–36 months (toddler RCT). Twenty-two local government areas (LGAs) in the state of Victoria were funded to provide these programs, which were established as new offerings within the LGAs’ suite of early childhood services. LGAs nominated service delivery locations which were then randomly allocated (within LGA) to provide *smalltalk group-only*, *smalltalk plus*, or an alternative group program with no *smalltalk* content. The current paper examines the two *smalltalk* arms of the trial (i.e. excluding the control group arm). *smalltalk* programs were delivered at 77 locations across the 22 LGAs: 38 locations provided *smalltalk group-only* and 39 locations provided *smalltalk plus*. Most locations employed a single facilitator who ran multiple consecutive groups.

### Participants

LGAs were responsible for parent recruitment (see Nicholson et al. (2016) for full details). Potential participants were identified through case finding (searching the MCH administrative database for eligible families), or referred into the program by MCH nurses or other relevant community workers. Self-referral was also encouraged via promotional materials in community agencies. Parents were eligible if they lived in the study area, had a child in the age range (6–12 months for infants; 12–36 months for toddler programs) and had at least one risk indicator of social disadvantage (i.e. on government benefits; young (< 25 years), single or socially isolated; low parent education; Indigenous or non-English speaking background).

**Fig. 1:**
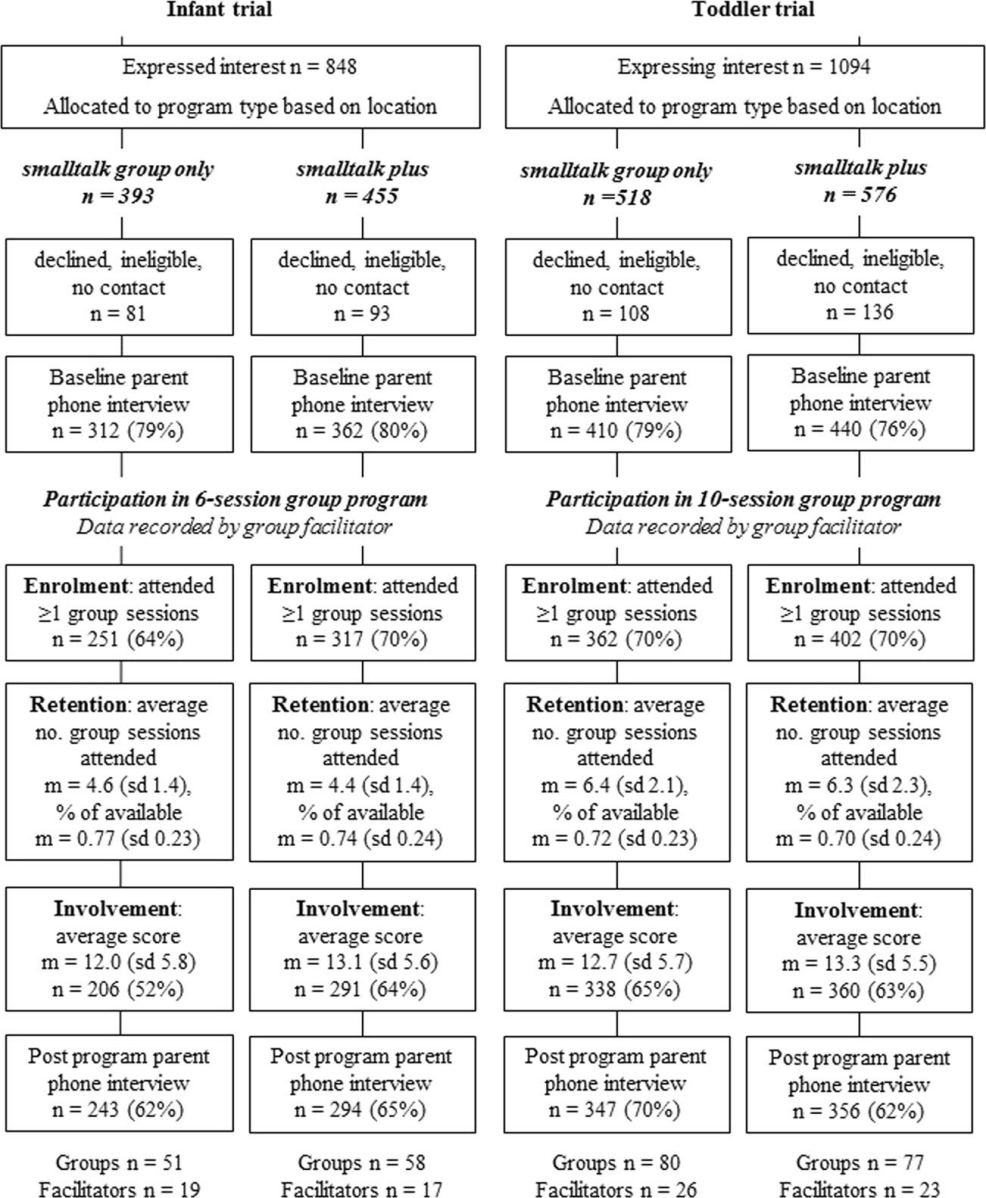
Participant flow for analyses of engagement

The contact details of families who expressed interest in participating were passed on to a local coordinator who contacted them to explain more about the program. Those who provided consent were then contacted by the researchers to undertake a baseline computer-assisted telephone interview (CATI) and in-home assessment, repeated 10 weeks later after program completion (see Fig. 1). Participants received a $50 gift voucher and a child’s book for each completed assessment. They were not paid for program attendance.

For the analyses of enrolment (see Fig. 1), the sample included all participants who consented to participate, were allocated to *smalltalk* and who provided baseline data (*N* = 1447; infant *n* = 629; toddler *n* = 818). For the analyses of retention and involvement (Fig. 1), the sample included all *smalltalk* participants with baseline data who attended at least one group session (*N* = 1332; infant *n* = 568; toddler *n* = 764).

### Measures

#### Participant Engagement

Parent engagement was recorded by group facilitators at the end of each group session using a purpose-designed booklet. Weekly attendance records were used to derive measures of *enrolment* defined as attending at least one group session (yes/no) and *retention* calculated as the proportion of sessions attended by the parent. *Involvement* was a summed score derived from facilitator records for each of six parenting strategies of whether the strategy was (i) discussed with the parent (‘exposure’), (ii) rehearsed by the parent in the group (‘application’) or (iii) practiced by the parent at home (‘generalisation’; rated yes/no; summed range 0–18).

#### Predictors of Engagement

Table 2 describes the four types of predictor measures collected. *Parent and family characteristics* were those factors specific to the family that could be assessed prior to commencement of the program and had the potential to impact on engagement. They included demographic factors, adverse life events, parenting self-efficacy, psychological distress and use of other services. These were assessed at baseline via a telephone interview.

**Table 2:** Measures of parent and family characteristics, participation barriers, program factors and contextual factors

Variable	Measure	Data collection	
		Source	Collected^a^
Parent and family factors			
Demographics	Parent and child age and gender, family structure and size, parent education, employment status, household income, government benefits, language spoken at home	Parent	Baseline
Life event stress	List of Threatening Experiences (LTE-Q): 7-item yes/no list of stressful life events in last 12 months (Brugha and Cragg 1990), e.g. “You had a major financial difficulty”. Total score 0 to 7	Parent	Baseline
Parent global self-efficacy	1 item on a 5-point scale, overall efficacy as a parent (Zubrick et al. 2014), “Overall, as a parent, do you feel that you are…?”, e.g. “a better than average parent”, producing a score between 1 and 5	Parent	Baseline
Psychosocial distress	Kessler-6 (K6): 6-item psychosocial screener on a 5-point scale assessing emotional distress in the last 4 weeks (Kessler et al. 2002). “About how often did you feel…?”, e.g. “nervous”. Total score 0 to 24	Parent	Baseline
Seeing other services	Use of early childhood services: 1 item “Have you or your child seen anyone other than your maternal and child health nurse in relation to your own health or your child’s health or behaviour?”	Parent	Baseline
Participation barriers			
Family	Family barriers to attendance: 4 yes/no items; e.g. difficulties with child health	Parent	Post
Logistical	Logistic barriers to attendance: 4 yes/no items; e.g. difficulties with transport	Parent	Post
Program	Program barriers to attendance: 3 yes/no items; e.g. difficulty relating to other parents in the group	Parent	Post
Program factors			
Group climate	6 items: level of rapport, time management, achievement of session goals, group cohesiveness, active participation by the group (5-point scale: 1 = much less than expected, 5 = much better than expected) and disruption of session by unanticipated events (5-point scale: 1 = none, 5 = extensive disruption)	Facilitator	Weekly
	Contact with parent between sessions: 1 item (yes/no)	Facilitator	Weekly
Facilitator	Demographics: age, gender, qualifications, experience in early childhood sector/parenting support	Facilitator	Pre-training
	Confidence in skills/competency: 6 skills, e.g. “Identifying specific needs of families” on a 5-point scale from 1 = ‘no level of skill/knowledge in the area’ to 5 = ‘advanced level of skill/knowledge’	Facilitator	Post-training
	Preparedness to deliver *smalltalk*: 5 items (e.g. How confident do you feel to be able to communicate the parenting strategies to parents?) 5-point scale (1 = not at all, 5 = very). Total score 5 to 25	Facilitator	Post-training
Contextual factors			
Area/population	Census of Population and Housing data (Australian Bureau of Statistics 2011) including geographic area (km^2^), population size and density; number of children 0–4 years; rate of language other than English spoken at home; rate of unemployment; number of households without a motor vehicle		
Neighbourhood disadvantage	Socio-Economic Indexes for Areas (SEIFA: Australian Bureau of Statistics 2011) Proportion of children in the ‘developmentally vulnerable’ range (lowest 10% nationally) for language, cognitive and general knowledge, Australian Early Development Census (AEDC 2015)		

^a^Weekly = recorded by facilitator at end of each group session; Baseline (*T* = 0); Post = after program completion (*T* = 12 weeks); Pre/post-training = immediately before/after facilitator training

*Participation barriers* were the parents’ subjective reports of the things that prevented or made it difficult for them to attend the program. These were assessed using a checklist of three types of common barriers (family, logistical and program-related), previously found to differentiate high and low attending families (Berthelsen et al. 2012). They were administered by telephone interview at the end of the program.

*Program factors* were assessed after each session using items developed for use in group parent interventions by facilitators from a variety of backgrounds (Berthelsen et al. 2012; Nicholson et al. 2010). Items rated on a five-point scale reflected the facilitator’s perceptions of how well each session went (‘session quality’) in terms of rapport established with the group, time management, achievement of session goals, group cohesion, overall active participation and disruptions to the session. Concordance with independent observer ratings (for a random 5% of sessions; *n* = 38) at ± 1 was > 89%. Facilitators also recorded whether they had contact with any family between sessions. Facilitator demographics, knowledge, confidence and preparedness to deliver the program were also conceptualised as program factors. These were assessed by facilitator questionnaires completed before and after training in program delivery. Session quality and facilitator characteristics assessed using these measures have been shown to impact on program outcomes (Nicholson et al. 2010) and levels of parent attendance (Berthelsen et al. 2012).

*Contextual factors* were assessed using publically available statistics. For each LGA, indicators of population vulnerability were extracted from data collected by the Australian Bureau of Statistics (ABS) and the Australian Early Development Census (AEDC 2015).

### Data Analysis

Statistical analyses were conducted using Stata version 13.1 (StataCorp, 2013). To identify predictors of participant engagement, multilevel models were used: logistic regression models for the analyses of enrolment (yes/no) and linear regression models for the analyses of retention and involvement (continuous scores). Models were stratified by infant and toddler platforms, with random intercepts, and accounted for the nesting of individuals within intervention groups and groups within facilitators. Multivariable analyses included participants with complete data resulting in reduced sample sizes as presented in the tables. Contextual factors were examined separately due to the small number of clusters. Unadjusted regression analyses were conducted on combined data from the infant and toddler platforms. Clustering of individuals within groups and groups within LGA (*N* = 22) was accounted for.

## Results

### Participant and Facilitator Characteristics

Parents who completed baseline measures (*N =* 1447) were predominantly mothers (98%), and ranged in age from 17 to 62 years (*M* = 32.4, *SD =* 6.1). There were similar numbers of male and female children in the sample (49% female), with a mean age of 8.0 months (*SD =* 2.2) in the infant trial and 22.6 months (*SD* = 7.2) in the toddler trial. Most children were from two-parent families (68% married; 21% de facto), with Australian-born parents (77%) who had completed year 12 or equivalent (87%). The majority (84%) experienced one or more risk factors for poor development (e.g. single parent, parental unemployment, low income).

Fifty-eight staff were trained as *smalltalk* facilitators. All were female, aged 23–59 years (*M* = 41.0, *SD =* 9.2), and the majority spoke English at home (81%). Twenty percent were certificate qualified; 40% had a university degree (in community service 50%; education 26%; health 11%; other 13%). Two thirds had previously run playgroups or parenting groups (67%).

### Predictors of Enrolment

The majority of parents who consented to participate and completed the baseline telephone interview went on to ‘enrol’ as defined by attending at least one group session (*n* = 568 of 629, 90% in the infant platform; *n* = 764 of 818, 93% in the toddler platform). Multilevel logistic regression models were used to identify parent/family factors associated with enrolment. Participation barriers and program factors were not included in the models, as parents could only be exposed to these after having attended the program. Unadjusted (univariate) models are available online (Supplemental Table 1). In the adjusted models, program type and two parent factors were significantly associated with enrolment. In the infant platform, allocation to *smalltalk plus* was higher amongst those who enrolled compared to those who did not (56 vs. 39%, OR = 2.28, 95% CI = 1.17–4.46, *p* = 0.016). A similar trend was evident in the toddler platform (53 vs. 39%, OR = 2.14, 95% CI = 0.98–4.67, *p* = 0.056). In the infant platform, young parenthood (15 vs. 46%, OR = 0.37, 95% CI = 0.17–0.79, *p* = 0.010) and receipt of government benefits as the main source of family income (16 vs. 53%, OR = 0.35, 95% CI = 0.13–0.94, *p* = 0.038) were lower amongst those who enrolled compared to those who did not. There were no significant parent/family predictors of enrolment in the toddler platform.

### Predictors of Retention

Parents attended an average of 4.5 sessions in the infant program and 6.4 sessions in the toddler program equating to 75 and 64% of offered sessions respectively. There were no differences for *group-only* vs. *smalltalk plus*. As shown in Table 3 (for unadjusted estimates, see Supplemental Table 2), retention to the group-only program in both the infant and toddler platforms was predominantly associated with program barriers including family-related (general family, parents’ own and child’s health/behaviour; infant platform only) and logistic difficulties (transport, fitting with child routine, medical appointments, work commitments; mostly both platforms) and believing the child was not benefiting (toddler platform only). Parent psychological distress was associated with lower enrolment for the toddler platform.

**Table 3:** Predictors of participant retention in *smalltalk group-only* and *smalltalk plus* for the infant and toddler platforms

	*smalltalk group-only*				*smalltalk group plus home coaching*			
	Infant (*n* = 231)		Toddler (*n* = 308)		Infant (*n* = 262)		Toddler (*n* = 328)	
	Coeff (95% CI)	*p*	Coeff (95% CI)	*p*	Coeff (95% CI)	*p*	Coeff (95% CI)	*p*
Parent and family factors								
No parent employed							0.12 (0.01, 0.23)	0.034
Government benefits							− 0.11 (− 0.20, − 0.02)	0.013
Life events					− 0.03 (− 0.07, − 0.002)	0.036		
Symptomatic for psychological distress			− 0.07 (− 0.14, − 0.001)	0.048				
Participation barriers								
Family-related difficulties								
General family difficulties	− 0.11 (− 0.19, − 0.02)	0.012			− 0.15 (− 0.28, − 0.02)	0.028		
Difficulties with own health	− 0.09 (− 0.17, − 0.01)	0.031						
Child’s health/behaviour	− 0.08 (− 0.13, − 0.02)	0.013					− 0.05 (− 0.10, − 0.01)	0.029
Logistic difficulties								
Transport to and from group			− 0.13 (− 0.26, − 0.01)	0.035			− 0.17 (− 0.27, − 0.07)	0.001
Fitting in with child’s routine	− 0.06 (− 0.11, − 0.01)	0.011	− 0.06 (− 0.12, − 0.005)	0.034				
Fitting in medical or other appointments	− 0.15 (− 0.23, − 0.07)	< 0.001	− 0.09 (− 0.16, − 0.02)	0.012				
Work commitments	− 0.14 (− 0.24, − 0.03)	0.009	− 0.14 (− 0.22, − 0.05)	0.001				
Program-related difficulties								
Did not believe child was benefiting			− 0.16 (− 0.25, − 0.08)	< 0.001				
Program factors								
Group cohesiveness					0.10 (0.01, 0.18)	0.027		
Contact with family between sessions							0.11 (0.02, 0.20)	0.012
Intraclass correlations	*ICC (95% CI)*		*ICC (95% CI)*		*ICC (95% CI)*		*ICC (95% CI)*	
Facilitator-level ICC	0.01 (0.00, 0.99)		0.03 (0.00, 0.32)		0.00 (0.00, 0.00)		0.04 (0.00, 0.34)	
Group-within facilitator-level ICC	0.11 (0.03, 0.34)		0.04 (0.00, 0.37)		0.06 (0.01, 0.28)		0.16 (0.07, 0.35)	

Data are unstandardised regression coefficients with 95% confidence intervals (CI) from multilevel linear regression analyses with random effects for group and facilitator. Intraclass correlations (ICC) with 95% CI are reported at the facilitator and group-within facilitator levels. Adjusted for other individual factors (parent age, family structure, parent education, low parenting efficacy), other participation barriers (care of other children, relating to other parents) and other program factors (group rapport, active participation, facilitator age) significant at *p* < .10 in unadjusted models

In contrast, retention in *smalltalk plus* was associated with a mix of parent/family factors, program barriers and program factors which differed for the two platforms. For the infant platform, retention was higher for families with fewer stressful life events, lower family-related difficulties and higher facilitator-rated group cohesiveness*.* For the toddler platform, retention was lower for those receiving government benefits, and families reporting child health/behavioural barriers or transport barriers, while having no parent employed and increased facilitator contact between sessions were associated with higher retention.

### Predictors of Involvement

As shown in Table 4 (for unadjusted estimates, see Supplemental Table 3), associations varied by program type and platform for involvement. For the group-only program in the infant platform, involvement was associated with three participation barriers and one program factor. Involvement was lower for those reporting difficulties with their health, appointments and work commitments. Involvement was also lower when the group facilitator was older. For the group-only program in the toddler platform, involvement was associated with one parent/family factor, two barriers and one program factor. Involvement was lower for those experiencing psychological distress, with work commitments and who did not believe their child was benefiting. It was higher for those whose facilitator was more knowledgeable in family support.

**Table 4:** Predictors of participant involvement in *smalltalk group-only* and *smalltalk plus* for the infant and toddler platforms

	*smalltalk group-only*				*smalltalk group plus home coaching*			
	Infant (*n* = 183)		Toddler (*n* = 301)		Infant (*n* = 264)		Toddler (*n* = 295)	
	Coeff (95% CI)	*p*	Coeff (95% CI)	*p*	Coeff (95% CI)	*p*	Coeff (95% CI)	*p*
Parent and family factors								
Parent age (less than or equal to 25 years)					− 1.46 (− 2.74, − 0.18)	0.026		
Language other than English					− 1.36 (− 2.57, − 0.14)	0.028		
Symptomatic for psychological distress			− 1.82 (− 2.86, − 0.78)	0.001				
Participation barriers								
Family-related difficulties								
Difficulties with own health	− 1.75 (− 3.29, − 0.21)	0.026						
Logistic difficulties								
Transport to and from group							− 2.20 (− 4.12, − 0.29)	0.024
Fitting in medical or other appointments	− 1.87 (− 3.42, − 0.33)	0.018						
Work commitments	− 2.51 (− 4.86, − 0.17)	0.036	− 1.86 (− 3.17, − 0.55)	0.006				
Program-related difficulties								
Did not believe child was benefiting			− 2.55 (− 3.85, − 1.25)	< 0.001				
Program factors								
Group cohesiveness					3.09 (0.36, 5.83)	0.027		
Contact with family between sessions							3.39 (0.85, 5.93)	0.009
Facilitator age	− 0.18 (− 0.33, − 0.03)	0.018						
Facilitator experience with groups					− 3.03 (− 5.71, − 0.35)	0.027		
Facilitator family support knowledge/skills			0.45 (0.14, 0.76)	0.005				
Intraclass correlations	*ICC (95% CI)*		*ICC (95% CI)*		*ICC (95% CI)*		*ICC (95% CI)*	
Facilitator-level ICC	0.14 (0.02, 0.62)		0.22 (0.07, 0.52)		0.03 (0.00, 0.97)		0.29 (0.10, 0.60)	
Group-within facilitator-level ICC	0.61 (0.45, 0.74)		0.70 (0.59, 0.79)		0.50 (0.36, 0.64)		0.71 (0.58, 0.81)	

Data are unstandardised regression coefficients with 95% confidence intervals (CI) from multilevel linear regression analyses with random effects for group and facilitator. Intraclass correlations (ICC) with 95% CI are reported at the facilitator and group-within facilitator levels. Adjusted for other individual factors (child age, parent education, employment, government benefits, life events), other participation barriers (general family-related, child health/behaviour, transport, child routine) and other program factors (active participation, disruptions, facilitator early childhood experience) significant at *p* < .10 in unadjusted models

For the *smalltalk plus* programs in the infant platform, involvement was associated with two parent/family factors and two program factors. Involvement was lower for those who were young parents or had a main language other than English. Involvement was higher for groups with higher group cohesiveness, but unexpectedly was lower with facilitators who had more experience with playgroups/parent groups. For *smalltalk plus* in the toddler platform, involvement was associated with only one barrier (lower transport difficulties) and one program factor (greater facilitator contact between sessions).

For involvement, intraclass correlations (ICC) were high, particularly at the group level (range 0.50 to 0.71), indicating that participants within the same group were highly correlated with respect to their involvement. This contrasts with retention where there were lower levels of clustering within groups (range 0.00–0.04) and facilitators (range 0.04–0.16).

### Community-Level Contextual Predictors

To examine community-level contextual factors, data were combined across the infant and toddler platforms. Lower enrolment was associated with higher community levels of long-term parent unemployment (OR = 0.80; 95% CI = 0.67–0.96; *p* = 0.015) and higher community rates of child language and cognitive vulnerability (OR = 0.87; 95% CI = 0.79–0.95, *p* = 0.001). Higher enrolment was associated with greater neighbourhood socio-economic advantage (SEIFA score divided by 100; OR = 2.17; 95% CI = 1.18–3.99; *p* = 0.013). No associations were found between community factors and the measures of parent retention or parent involvement.

## Discussion

*smalltalk* was designed as a preventive group program for vulnerable families with young children. In a large-scale cluster randomised controlled trial, we were broadly successful in engaging with our targeted sample. Of those parents who expressed interest in the program, 64 to 70% attended with families attending an average of 63 to 77% of sessions (around 4.5 sessions for those in the 6-session infant programs; just over 6 sessions for those in the 10-session toddler programs). These rates compare favourably with other approaches which have reported poorer enrolment (Baker et al. 2011; Garvey et al. 2006) and higher dropout (Axford et al. 2012; Morawska and Sanders 2006b). In this paper, we sought to identify the individual, program and contextual factors that were associated with differing levels of enrolment, retention and involvement in the *smalltalk* programs. Study findings support a multidimensional model of parental engagement with different sets of predictors identified for each measure of engagement. We also found differences between the group-only and *smalltalk plus* programs and for the infant compared to toddler platforms.

For enrolment, we hypothesised that parent and family disadvantage would be associated with lower enrolment as shown in other studies. There was limited evidence for this. In the infant platform only, enrolment was lower for young parents and for parents receiving government benefits. Enrolment in the toddler platform was not associated with these factors, and there was no evidence in either platform of adverse enrolment effects from a range of other parent/family factors identified in previous studies including parental unemployment, low education or cultural and linguistic diversity (Brown et al. 2012; Eisner and Meidert 2011; Heinrichs et al. 2005; Mendez et al. 2009; Sanders and Kirby 2012). Overall, these data indicate that a mostly representative sample of the families who expressed interest in the *smalltalk* programs made the transition from recruitment to enrolment.

The lower enrolment by young parents and those on government benefits in the infant platform suggests some unique challenges for these parents that were not evident in the toddler platform. There are several possibilities. This difference could reflect how difficult it is for parents of very young children, despite their best intentions, to attend appointments outside the home. Additionally, the infant *smalltalk* intervention was delivered as a structured group program with a focus on parenting support. Parents who inadvertently missed the first session may have felt that this would limit the value of subsequent sessions. For vulnerable parents who were still adjusting to the demands of parenthood, it is also possible that referral into such a program was both practically challenging and potentially perceived as stigmatising or threatening (Bussing et al. 2003; Cortis et al. 2009). In contrast, the toddler *smalltalk* programs were delivered in the less formal supported playgroup setting, with an ‘overt’ focus on play and social interaction and a more fluid approach to attendance (missed sessions are an expected norm). This may offer a ‘softer’ less threatening entry point, allowing parents to feel that they can turn up and see what it is like without necessarily committing to the full program.

Families recruited to home coaching programs (*smalltalk plus*) were more than twice as likely to proceed to enrolment than those allocated to the group-only condition, even though the home coaching had not yet started. *smalltalk plus* parents had a research assessment conducted by their home coach prior to starting the group; this was conducted by research staff for group-only parents. It is likely this initial contact with the home coach served as an ‘ice breaker’ to program commencement.

We predicted that once parents had attended *smalltalk*, parent/family factors would not be associated with retention and involvement, while participation barriers, program and community factors would. There was some support for this hypothesis. Across the multivariable analyses, relatively few parent/family factors were associated with retention or involvement. Participation barriers due to family (parent health or child health/behaviour), logistic (conflicting appointments, work, lack of transport) and the program (did not believe child was benefiting) difficulties were associated with lower retention and involvement. This was most evident for retention in the group-only programs (both platforms) and to a lesser extent, involvement. This highlights the compound nature of the multiple challenges facing disadvantaged families and lends weight to the argument that in addition to some *families* being hard to reach, some *programs* are hard for families to access (Cortis et al. 2009).

The pattern of factors associated with our measures of parent engagement varied between the group-only programs and those that included home coaching. In particular, the lack of associations between most participation barriers and the measures of retention and involvement for the *smalltalk plus* programs suggest that having a home coach may have helped parents to overcome these barriers. However, paradoxically, group cohesiveness and facilitator follow-up between sessions were associated with better retention and involvement for families receiving home coaching, but not those in the group-only condition. Reasons for this are not immediately clear. One possibility is that *smalltalk plus* parents, who were already receiving program content via home coaching, felt less need to persist in attending and actively engaging within the group sessions when the group climate or facilitation was sub-optimal.

Previous studies have highlighted the importance of a skilled workforce for engaging families (Whittaker and Cowley 2010). In this study, facilitator factors were not associated with retention, but were associated with parents’ active involvement during group sessions. While our findings showed some support for the benefits of facilitator skills and knowledge in the toddler group-only programs, contrary to expectations, for the parents of infants, older facilitator age (group-only programs) and greater experience (*smalltalk plus* programs) predicted lower levels of active parent involvement. It appears that an older facilitator did not deter parents from attending, but may have made them reluctant to practice skills in the group setting. It is also possible that older facilitators are accustomed to more didactic methods of instruction which allow less opportunity for active parent participation.

A novel aspect of this study was the inclusion of community and contextual factors in the model. As was the case with individual indicators of disadvantage, there was some evidence that parents from areas with higher levels of unemployment and higher indices of area disadvantage and child vulnerability were less likely to attend groups. These contextual factors were not predictive of subsequent retention or involvement, suggesting that once parents from more disadvantaged areas attend, they continue to do so and are as involved as other parents.

This study had several limitations including program design features that precluded us being able to measure the number of parents approached by LGA staff for potential recruitment, meaning that enrolment analyses only included those who had signed up and completed baseline measures potentially diluting effects of parent/family factors on enrolment. Our measure of parent involvement was derived from facilitator records monitoring how much of the intervention their group members were receiving. The approach was not only designed to aid program delivery but also relied upon parents’ sharing and accurate reporting of home practice. Findings from this measure should therefore be viewed with caution. Future studies using validated observational measures are needed to determine whether the patterns reported here are robust. Our analyses of the effects of community-level factors are also best regarded as exploratory. These data were reported at the LGA level, with the small number of LGAs precluding their inclusion in multivariate models. Finally, data reported here are from a larger project, in which participants were reimbursed for data collection time. While reimbursements were not related to program attendance, other studies who they can have a powerful impact on program engagement (Heinrichs and Jensen-Doss 2010; Ingoldsby 2010). It is feasible that the reimbursements impacted on parent’s engagement decisions for this study.

### Conclusions and Implications

Parenting programs can only be effective if they reach and engage the populations they are designed for. This is an ongoing challenge for the early childhood sector, particularly when programs focus on prevention of a problem that has not yet occurred (Garvey et al. 2006; Whittaker and Cowley 2010). By taking an ecological approach that considered individual, program and contextual factors, we identified risk factors for poor engagement at each stage of service delivery (enrolment, retention and involvement), providing insight into the types of supports services need to provide to reach and engage the communities they serve.

Our findings suggest that the enrolment of vulnerable parents in preventively focused interventions can be strong when the programs are offered as part of existing and respected service platforms which are known to be universally available. Inclusion of a structured curriculum in a program that is oriented towards providing play and social opportunities was associated with better enrolment that the more typical structured parenting group, and this approach is now being used as the standard method for delivering *smalltalk* programs including to parents of infants. This uniquely Australian approach warrants consideration elsewhere.

Providing a home coach may assist vulnerable parents to identify and overcome some of the practical barriers that would otherwise prevent continued group attendance. However, it is also important to maintain a focus on quality group facilitation processes, as our data suggest that parents who have a home coach may be more likely to drop out and disengage when their group program is poorly facilitated. Our parent involvement results also highlight the need to ensure that all group facilitators are equally skilled in supporting parents’ active participation. Finally, our community-level analyses indicate that population indices of disadvantage can provide services with valuable information as to where additional supports are required to ensure that services are received by those families who need them most. Given that community-level factors were most strongly associated with enrolment, if services are able to encourage vulnerable families to attend at least once, then this type of program is effective for retaining and involving them. Previous research has largely conceptualised engagement as a proxy for ‘dose’ with the assumption that higher engagement will lead to more positive program outcomes (Brown et al. 2012; Whittaker and Cowley 2010). Future research to determine which aspects of engagement are most closely linked to outcomes will further assist services to direct limited resources more effectively.

This study has underscored some of the common difficulties in engaging parents from disadvantaged families in preventive interventions and highlighted some contexts that improve engagement. Each family has a unique context that shapes their ability to engage with services. Service providers need to be mindful of the multiple demands placed on parents and that the factors that get in the way for families ‘getting to programs’ may be different to what ‘keeps them coming’ and ‘how much’ they engage once they are there. There are implications in terms of service costs and workforce planning. While home coaching appears to support families’ engagement, this is a costly service that is difficult to implement and sustain outside of well-funded initiatives. It requires a skilled workforce provided with quality training and ongoing professional support. In contrast, family services in Australia are often funded through short-term schemes, with a highly casualised workforce. Staff turnover can pose significant challenges for quality, consistency and fidelity of service provision (Gomby 2007; Olds et al. 2015). To actively engage disadvantaged families in these programs, greater consideration is also required on how to ensure an appropriately trained and supported stable workforce.

## Electronic Supplementary Material

Supplemental Table 1 (DOCX 23 kb)

Supplemental Table 2 (DOCX 33 kb)

Supplemental Table 3 (DOCX 34 kb)

## References

[CR1] Australian Bureau of Statistics. (2011). Census of Population and Housing: Socio-Economic Indexes for Areas (SEIFA), Australia, 2011 (cat. no. 2033.0.55.001). Retrieved from http://www.abs.gov.au/census. Accessed 12 Dec 2016.

[CR2] Australian Early Development Census. (2015). Retrieved from https://www.aedc.gov.au/. Accessed 1 Dec 2016.

[CR3] Axford N, Lehtonen M, Kaoukji D, Tobin K, Berry V (2012). Engaging parents in parenting programs: Lessons from research and practice. Children and Youth Services Review.

[CR4] Baker CN, Arnold DH, Meagher S (2011). Enrollment and attendance in a parent training prevention program for conduct problems. Prevention Science.

[CR5] Berthelsen, D., Williams, K., Abad, V., Vogel, L., & Nicholson, J. (2012). *The parents at playgroup research report: Engaging families in supported playgroups*. Retrieved from http://eprints.qut.edu.au/50875/1/Parents_at_Playgroup_Final_Report.pdf. Accessed 12 June 2014.

[CR6] Bradley RH, Corwyn RF (2002). Socioeconomic status and child development. Annual Review of Psychology.

[CR7] Brown LD, Goslin MC, Feinberg ME (2012). Relating engagement to outcomes in prevention: The case of a parenting program for couples. American Journal of Community Psychology.

[CR8] Brugha T, Cragg D (1990). The list of threatening experiences: The reliability and validity of a brief life events questionnaire. Acta Psychiatrica Scandinavica.

[CR9] Bussing R, Zima BT, Gary FA, Garvan CW (2003). Barriers to detection, help-seeking, and service use for children with ADHD symptoms. The Journal of Behavioural Health Services & Research.

[CR10] Cortis, N., Katz, I., & Patulny, R. (2009). *Engaging hard-to-reach families and children* (Occasional Paper No. 26). Retrieved from Canberra.

[CR11] Eisner M, Meidert U (2011). Stages of parental engagement in a universal parent training program. Journal of Primary Prevention.

[CR12] Garvey C, Julion W, Fogg L, Kratovil A, Gross D (2006). Measuring participation in a prevention trial with parents of young children. Research in Nursing & Health.

[CR13] Gomby DS (2007). The promise and limitations of home visiting: Implementing effective programs. Child Abuse & Neglect.

[CR14] Gross D, Julion W, Fogg L (2001). What motivates participation and dropout among low-income urban families of color in a prevention intervention?. Family Relations.

[CR15] Hackworth N. J., Berthelsen D., Matthews J., Westrupp E. M., Cann W., Ukoumunne O. C., Bennetts S. K., Phan T., Scicluna A., Trajanovska M., Yu M., Nicholson J. M. (2017). Impact of a Brief Group Intervention to Enhance Parenting and the Home Learning Environment for Children Aged 6–36 Months: a Cluster Randomised Controlled Trial. Prevention Science.

[CR16] Hart B, Risley TR (1995). Meaningful differences in the everyday experience of young American children.

[CR17] Heinrichs N, Jensen-Doss A (2010). The effects of incentives on families’ long-term outcome in a parenting program. Journal of Clinical Child & Adolescent Psychology.

[CR18] Heinrichs N, Bertram H, Kuschel A, Hahlweg K (2005). Parent recruitment and retention in a universal prevention program for child behavior and emotional problems: Barriers to research and program participation. Prevention Science.

[CR19] Ingoldsby E (2010). Review of interventions to improve family engagement and retention in parent and child mental health programs. Journal of Child & Family Studies.

[CR20] KESSLER R.??C., ANDREWS G., COLPE L.??J., HIRIPI E., MROCZEK D.??K., NORMAND S.-L.??T., WALTERS E.??E., ZASLAVSKY A.??M. (2002). Short screening scales to monitor population prevalences and trends in non-specific psychological distress. Psychological Medicine.

[CR21] Landry SH, Smith KE, Swank PR, Guttentag C (2008). A responsive parenting intervention: The optimal timing across early childhood for impacting maternal behaviors and child outcomes. Developmental Psychology.

[CR22] Matthews, J., Cameron, E., Fox, S., Hackworth, N., Kitanovski, M., & Vista, A. (2011). Supported playgroups and parent groups initiative (SPPI) process evaluation. Retrieved from Department of Education and Early Childhood Development website: http://www.education.vic.gov.au/Documents/about/programs/health/sppiprocesseval.pdf. Accessed 12 Jun 2014.

[CR23] McCurdy K, Daro D (2001). Parent involvement in family support programs: An integrated theory. Family Relations.

[CR24] Mendez JL, Carpenter JL, LaForett DR, Cohen JS (2009). Parental engagement and barriers to participation in a community-based preventive intervention. American Journal of Community Psychology.

[CR25] Miller GE, Prinz RJ (2003). Engagement of families in treatment for childhood conduct problems. Behavior Therapy.

[CR26] Morawska A, Sanders MR (2006). A review of parental engagement in parenting interventions and strategies to promote it. Journal of Children’s Services.

[CR27] Morawska A, Sanders MR (2006). Self-administered behavioural family intervention for parents of toddlers: Effectiveness and dissemination. Behaviour Research and Therapy.

[CR28] Nicholson JM, Berthelsen D, Williams K, Abad V (2010). National study of an early parenting intervention: Implementation differences on parent and child outcomes. Prevention Science.

[CR29] Nicholson, J. M., Cann, W., Matthews, J., Berthelsen, D., Ukoumunne, O. C., Trajanovska, M., … Westrupp, E. (2016). Enhancing the early home learning environment through a brief group parenting intervention: Study protocol for a cluster randomised controlled trial. *BMC Pediatrics, 16*(1), 1. 10.1186/s12887-016-0610-1. 10.1186/s12887-016-0610-1 PMC4890293 27255588

[CR30] Olds David L., Baca Pilar, McClatchey Maureen, Ingoldsby Erin M., Luckey Dennis W., Knudtson Michael D., Loch Joan M., Ramsey Mildred (2015). Cluster Randomized Controlled Trial of Intervention to Increase Participant Retention and Completed Home Visits in the Nurse-Family Partnership. Prevention Science.

[CR31] Sanders MR, Kirby JN (2012). Consumer engagement and the development, evaluation, and dissemination of evidence-based parenting programs. Behavior Therapy.

[CR32] Spoth R, Goldberg C, Redmond C (1999). Engaging families in longitudinal preventive intervention research: Discrete-time survival analysis of socioeconomic and social-emotional risk factors. Journal of Consulting and Clinical Psychology.

[CR33] Spoth R, Redmond C, Shin C (2000). Modeling factors influencing enrollment in family-focused preventive intervention research. Prevention Science.

[CR34] StataCorp (2013). Stata statistical software: Release 13.

[CR35] Victorino, C. C., & Gauthier, A. H. (2009). The social determinants of child health: Variations across health outcomes—A population-based cross-sectional analysis. *BMC Pediatrics, 9*. 10.1186/1471-2431-9-53. 10.1186/1471-2431-9-53 PMC2734529 19686599

[CR36] Whittaker KA, Cowley S (2010). An effective programme is not enough: A review of factors associated with poor attendance and engagement with parenting support programmes. Children and Society.

[CR37] Zubrick SR, Lucas N, Westrupp EM, Nicholson JM (2014). Parenting measures in the Longitudinal Study of Australian Children: Construct validity and measurement quality, Waves 1 to 4. (LSAC Technical Paper No. 12).

