# Supplementary material for: What Influences Parental Engagement in Early Intervention? Parent, Program and Community Predictors of Enrolment, Retention and Involvement
Source: Prev Sci. 2018 Apr 9;19(7):880–93. doi: 10.1007/s11121-018-0897-2 (PMC6182377; doi:10.1007/s11121-018-0897-2)
Supplement: Supplementary file 1 — (DOCX 23 kb) [file 11121_2018_897_MOESM1_ESM.docx]

*Supplemental Table 1:* Parent and family predictors of participant enrolment: showing regression coefficients from unadjusted logistic regression models, for the infant and toddler platforms (n=1447).

|  | Infant platform (n=629) | | Toddler platform (n=818) | |
| --- | --- | --- | --- | --- |
|  | OR (95% CI) | p | OR (95% CI) | p |
| Child age (in months) | 0.87 (0.77, 0.99) | 0.031 | 1.00 (0.96, 1.04) | 0.893 |
| Parent age (less than or equal to 25 years) | 0.21 (0.12, 0.38) | <0.001 | 0.26 (0.12, 0.55) | <0.001 |
| Single parent | 0.30 (0.16, 0.57) | <0.001 | 0.28 (0.13, 0.58) | 0.001 |
| Parent education (year 12 or less) | 0.44 (0.24, 0.82) | 0.010 | 0.50 (0.26, 0.95) | 0.033 |
| Language other than English | 0.84 (0.37, 1.93) | 0.684 | 0.70 (0.37, 1.31) | 0.259 |
| Government benefit | 0.18 (0.10, 0.31) | <0.001 | 0.29 (0.15, 0.54) | <0.001 |
| Parent employment |  |  |  |  |
| Both parents employed | 3.34 (1.60, 6.99) | 0.001 | 2.06 (1.02, 4.17) | 0.044 |
| No parent employed | 0.17 (0.07, 0.39) | <0.001 | 0.49 (0.17, 1.38) | 0.177 |
| 2 or more children in household | 0.87 (0.50, 1.52) | 0.635 | 0.61 (0.33, 1.12) | 0.111 |
| Life event stress | 0.66 (0.51, 0.86) | 0.002 | 0.68 (0.53, 0.88) | 0.004 |
| Global low self-efficacy | 0.58 (0.33, 1.02) | 0.060 | 0.93 (0.50, 1.74) | 0.821 |
| Symptomatic for psychological distress | 0.28 (0.13, 0.58) | 0.001 | 0.74 (0.35, 1.59) | 0.444 |
| Seeing other services | 1.32 (0.56, 3.14) | 0.530 | 0.54 (0.22, 1.38) | 0.199 |

*Note:* Table shows odds ratios (OR) with 95% confidence intervals (CI)
