# Supplementary material for: What Influences Parental Engagement in Early Intervention? Parent, Program and Community Predictors of Enrolment, Retention and Involvement
Source: Prev Sci. 2018 Apr 9;19(7):880–93. doi: 10.1007/s11121-018-0897-2 (PMC6182377; doi:10.1007/s11121-018-0897-2)
Supplement: Supplementary file 2 — (DOCX 33 kb) [file 11121_2018_897_MOESM2_ESM.docx]

*Supplemental Table 2:* Parent and family and program predictors of participant retention in the parenting interventions *smalltalk group-only* and *smalltalk plus*: showing regression coefficients from unadjusted linear regression models, for the infant and toddler platforms (n=1332).

|  | Infant platform | | | | Toddler platform | | | |
| --- | --- | --- | --- | --- | --- | --- | --- | --- |
|  | *group-only* (n=251) | | *smalltalk plus* (n=317) | | *group-only* (n=362) | | *smalltalk plus* (n=402) | |
|  | Coeff (95% CI) | p | Coeff (95% CI) | p | Coeff (95% CI) | p | Coeff (95% CI) | p |
| **Parent and family factors** |  |  |  |  |  |  |  |  |
| Child age (months) | -0.01 (-0.02, 0.01) | 0.468 | -0.01 (-0.02, 0.00) | 0.159 | 0.00 (0.00, 0.00) | 0.400 | 0.00 (0.00, 0.00) | 0.681 |
| Parent age (less than or equal to 25) | -0.13 (-0.22, -0.05) | 0.003 | -0.16 (-0.22, -0.09) | <0.001 | -0.12 (-0.21, -0.03) | 0.008 | -0.05 (-0.14, 0.03) | 0.219 |
| Single parent | -0.11 (-0.21, -0.02) | 0.023 | -0.07 (-0.15, 0.02) | 0.115 | -0.07 (-0.15, 0.02) | 0.122 | -0.04 (-0.12, 0.04) | 0.320 |
| Parent education (year 12 or less) | -0.06 (-0.12, 0.00) | 0.037 | -0.07 (-0.12, -0.02) | 0.011 | -0.07 (-0.11, -0.02) | 0.006 | -0.03 (-0.08, 0.02) | 0.266 |
| Language other than English | 0.02 (-0.08, 0.11) | 0.751 | -0.04 (-0.12, 0.04) | 0.286 | 0.02 (-0.04, 0.07) | 0.548 | 0.01 (-0.04, 0.07) | 0.640 |
| Government benefit | -0.06 (-0.14, 0.02) | 0.151 | -0.10 (-0.17, -0.03) | 0.004 | -0.12 (-0.19, -0.06) | <0.001 | -0.09 (-0.15, -0.02) | 0.007 |
| Parent employment |  |  |  |  |  |  |  |  |
| Both parents employed | 0.04 (-0.02, 0.10) | 0.211 | 0.03 (-0.03, 0.08) | 0.344 | 0.03 (-0.02, 0.08) | 0.183 | 0.04 (-0.01, 0.09) | 0.099 |
| No parent employed | 0.02 (-0.18, 0.22) | 0.871 | -0.17 (-0.29, -0.05) | 0.005 | -0.15 (-0.26, -0.04) | 0.009 | -0.06 (-0.16, 0.04) | 0.229 |
| 2 or more children in household | -0.01 (-0.07, 0.05) | 0.676 | 0.02 (-0.03, 0.08) | 0.419 | 0.01 (-0.04, 0.06) | 0.706 | 0.00 (-0.05, 0.04) | 0.847 |
| Life event stress | -0.04 (-0.07, 0.00) | 0.044 | -0.03 (-0.06, -0.01) | 0.019 | -0.04 (-0.07, -0.02) | 0.001 | -0.01 (-0.04, 0.01) | 0.332 |
| Global low self-efficacy | 0.00 (-0.06, 0.07) | 0.951 | -0.01 (-0.07, 0.05) | 0.745 | -0.06 (-0.12, -0.01) | 0.013 | 0.03 (-0.02, 0.08) | 0.282 |
| Symptomatic for psychological distress | -0.14 (-0.27, -0.02) | 0.020 | -0.04 (-0.13, 0.05) | 0.428 | -0.08 (-0.15, -0.01) | 0.025 | -0.02 (-0.08, 0.04) | 0.552 |
| Seeing other services | 0.05 (-0.06, 0.15) | 0.373 | -0.01 (-0.10, 0.08) | 0.795 | 0.00 (-0.07, 0.07) | 0.966 | 0.00 (-0.06, 0.06) | 0.954 |
| **Participation barriers** |  |  |  |  |  |  |  |  |
| *Family-related difficulties* |  |  |  |  |  |  |  |  |
| General family difficulties | -0.18 (-0.27, -0.10) | <0.001 | -0.13 (-0.25, -0.01) | 0.037 | -0.18 (-0.27, -0.10) | <0.001 | -0.05 (-0.13, 0.03) | 0.217 |
| Difficulties with own health | -0.16 (-0.24, -0.08) | <0.001 | -0.10 (-0.18, -0.02) | 0.012 | -0.08 (-0.14, -0.03) | 0.005 | -0.01 (-0.06, 0.05) | 0.850 |
| Child’s health/behaviour | -0.14 (-0.20, -0.09) | <0.001 | -0.04 (-0.10, 0.01) | 0.131 | -0.09 (-0.14, -0.05) | <0.001 | -0.06 (-0.10, -0.01) | 0.012 |
| Caring for other children | -0.07 (-0.15, 0.01) | 0.078 | 0.00 (-0.08, 0.08) | 0.970 | -0.06 (-0.12, 0.01) | 0.078 | -0.04 (-0.10, 0.02) | 0.182 |
| *Logistical difficulties* |  |  |  |  |  |  |  |  |
| Transport to and from group | -0.21 (-0.41, -0.01) | 0.040 | -0.29 (-0.41, -0.16) | <0.001 | -0.18 (-0.30, -0.06) | 0.003 | -0.19 (-0.29, -0.08) | 0.001 |
| Fitting in with child’s routine | -0.08 (-0.14, -0.03) | 0.002 | -0.05 (-0.10, 0.00) | 0.049 | -0.09 (-0.15, -0.03) | 0.004 | -0.05 (-0.10, 0.01) | 0.100 |
| Fitting in medical or other appointments | -0.21 (-0.29, -0.12) | <0.001 | -0.12 (-0.22, -0.03) | 0.010 | -0.12 (-0.19, -0.05) | 0.001 | -0.05 (-0.12, 0.01) | 0.125 |
| Work commitments | -0.17 (-0.29, -0.06) | 0.003 | -0.09 (-0.20, 0.02) | 0.094 | -0.16 (-0.24, -0.08) | <0.001 | -0.03 (-0.12, 0.06) | 0.539 |
| *Program-related difficulties* |  |  |  |  |  |  |  |  |
| Relating to other parents | -0.01 (-0.12, 0.10) | 0.859 | -0.09 (-0.18, 0.00) | 0.044 | -0.10 (-0.19, -0.02) | 0.014 | -0.07 (-0.14, 0.00) | 0.057 |
| Relating to staff | 0.19 (-0.21, 0.59) | 0.361 | -0.13 (-0.41, 0.15) | 0.371 | 0.03 (-0.15, 0.20) | 0.741 | 0.01 (-0.16, 0.19) | 0.884 |
| Did not believe child was benefiting | -0.14 (-0.25, -0.03) | 0.012 | -0.11 (-0.21, 0.00) | 0.044 | -0.17 (-0.25, -0.09) | <0.001 | -0.01 (-0.11, 0.08) | 0.765 |
| **Program factors** |  |  |  |  |  |  |  |  |
| *Group Climate* |  |  |  |  |  |  |  |  |
| Group rapport | 0.06 (-0.02, 0.13) | 0.136 | 0.09 (0.01, 0.17) | 0.024 | 0.01 (-0.05, 0.07) | 0.652 | 0.06 (-0.01, 0.13) | 0.083 |
| Time management | -0.01 (-0.10, 0.09) | 0.886 | 0.03 (-0.09, 0.15) | 0.664 | 0.02 (-0.04, 0.09) | 0.446 | 0.03 (-0.04, 0.10) | 0.353 |
| Session planning | 0.05 (-0.03, 0.14) | 0.227 | 0.05 (-0.04, 0.15) | 0.253 | 0.01 (-0.05, 0.07) | 0.753 | 0.03 (-0.04, 0.09) | 0.413 |
| Group cohesiveness | 0.05 (0.00, 0.11) | 0.048 | 0.12 (0.06, 0.17) | <0.001 | 0.05 (0.00, 0.10) | 0.040 | 0.06 (0.01, 0.11) | 0.018 |
| Active participation | 0.01 (-0.04, 0.07) | 0.626 | 0.09 (0.04, 0.15) | <0.001 | 0.03 (-0.03, 0.08) | 0.358 | 0.04 (-0.01, 0.10) | 0.106 |
| Unanticipated events | -0.02 (-0.07, 0.03) | 0.382 | -0.02 (-0.07, 0.03) | 0.493 | 0.01 (-0.05, 0.06) | 0.815 | -0.01 (-0.07, 0.05) | 0.847 |
| Contact with family between sessions | -0.02 (-0.11, 0.07) | 0.678 | 0.09 (0.00, 0.18) | 0.057 | 0.01 (-0.08, 0.10) | 0.817 | 0.46 (0.35, 0.57) | <0.001 |
| *Facilitator Characteristics* |  |  |  |  |  |  |  |  |
| Age | 0.00 (-0.01, 0.00) | 0.237 | 0.00 (0.00, 0.00) | 0.943 | 0.00 (0.00, 0.01) | 0.021 | 0.00 (-0.01, 0.00) | 0.616 |
| Not university educated | 0.01 (-0.06, 0.09) | 0.771 | -0.01 (-0.07, 0.06) | 0.878 | -0.03 (-0.12, 0.06) | 0.529 | -0.02 (-0.08, 0.04) | 0.582 |
| Experience in early childcare | 0.00 (-0.01, 0.00) | 0.316 | 0.00 (0.00, 0.00) | 0.880 | 0.00 (0.00, 0.00) | 0.507 | 0.00 (0.00, 0.00) | 0.803 |
| Experience with playgroups/parent groups | 0.03 (-0.06, 0.12) | 0.550 | 0.01 (-0.06, 0.09) | 0.713 | 0.01 (-0.06, 0.08) | 0.858 | 0.00 (-0.06, 0.06) | 0.932 |
| Knowledge/Skills about family support | 0.00 (-0.01, 0.01) | 0.557 | 0.01 (-0.01, 0.02) | 0.284 | 0.00 (-0.01, 0.01) | 0.754 | 0.00 (-0.01, 0.01) | 0.645 |
| Training evaluation | -0.001 (-0.02, 0.02) | 0.930 | -0.002 (-0.03, 0.02) | 0.880 | 0.0004 (-0.02, 0.02) | 0.961 | -0.01 (-0.02, 0.01) | 0.480 |

*Note:* Table shows regression coefficients with 95% confidence intervals (CI)
