# Supplementary material for: What Influences Parental Engagement in Early Intervention? Parent, Program and Community Predictors of Enrolment, Retention and Involvement
Source: Prev Sci. 2018 Apr 9;19(7):880–93. doi: 10.1007/s11121-018-0897-2 (PMC6182377; doi:10.1007/s11121-018-0897-2)
Supplement: Supplementary file 3 — (DOCX 34 kb) [file 11121_2018_897_MOESM3_ESM.docx]

*Supplemental Table 3:* Parent and family and program predictors of participant involvement in the parenting intervention *smalltalk group-only*  and *smalltalk plus*: showing regression coefficients from unadjusted linear regression models, for the toddler platform (n=1332).

|  | Infant platform | | | | Toddler platform | | | |
| --- | --- | --- | --- | --- | --- | --- | --- | --- |
|  | *group-only* (n=251) | | *smalltalk plus* (n=317) | | *group-only* (n=362) | | *smalltalk plus* (n=402) | |
|  | Coefficient  (95% CI) | p | Coeff (95% CI) | p | Coeff (95% CI) | p | Coeff (95% CI) | p |
| **Parent and family factors** |  |  |  |  |  |  |  |  |
| Child age (in months) | -0.33 (-0.59, -0.06) | 0.016 | -0.07 (-0.29, 0.15) | 0.538 | -0.03 (-0.09, 0.02) | 0.258 | 0.03 (-0.05, 0.10) | 0.481 |
| Parent age (less than or equal to 25) | -1.42 (-3.07, 0.24) | 0.094 | -2.15 (-3.51, -0.78) | 0.002 | 0.41 (-1.30, 2.13) | 0.637 | -1.17 (-3.04, 0.70) | 0.219 |
| Single parent | -1.34 (-3.12, 0.43) | 0.137 | -1.16 (-2.74, 0.41) | 0.148 | 0.33 (-1.10, 1.77) | 0.649 | -0.64 (-2.22, 0.95) | 0.431 |
| Parent education (year 12 or less) | -1.28 (-2.35, -0.21) | 0.019 | -1.03 (-2.03, -0.03) | 0.043 | -0.12 (-0.99, 0.75) | 0.780 | -0.10 (-1.13, 0.94) | 0.856 |
| Language other than English | -0.97 (-2.68, 0.73) | 0.263 | -1.30 (-2.69, 0.10) | 0.068 | -0.45 (-1.40, 0.49) | 0.345 | 0.53 (-0.68, 1.74) | 0.388 |
| Government benefit | -1.25 (-2.69, 0.18) | 0.087 | -1.31 (-2.71, 0.09) | 0.066 | -0.38 (-1.58, 0.81) | 0.529 | -1.38 (-2.65, -0.11) | 0.033 |
| Parent employment |  |  |  |  |  |  |  |  |
| Both parents employed | 0.71 (-0.38, 1.80) | 0.204 | 0.37 (-0.66, 1.39) | 0.485 | -0.41 (-1.28, 0.46) | 0.358 | 0.89 (-0.10, 1.89) | 0.078 |
| No parent employed | -2.56 (-6.19, 1.07) | 0.167 | -1.77 (-4.35, 0.80) | 0.177 | -1.28 (-3.27, 0.70) | 0.204 | -1.82 (-3.72, 0.09) | 0.062 |
| 2 or more children in household | -0.25 (-1.36, 0.85) | 0.653 | 0.11 (-0.92, 1.13) | 0.837 | 0.05 (-0.79, 0.88) | 0.913 | 0.06 (-0.94, 1.06) | 0.910 |
| Life event stress | -0.42 (-1.08, 0.25) | 0.218 | -0.49 (-1.05, 0.06) | 0.083 | -0.27 (-0.71, 0.18) | 0.245 | -0.28 (-0.75, 0.19) | 0.238 |
| Global low self-efficacy | 0.18 (-1.03, 1.39) | 0.767 | -0.40 (-1.50, 0.70) | 0.478 | 0.07 (-0.85, 0.99) | 0.879 | -0.56 (-1.52, 0.41) | 0.256 |
| Symptomatic for psychological distress | -2.23 (-4.34, -0.13) | 0.038 | 1.25 (-0.41, 2.90) | 0.140 | -1.48 (-2.77, -0.19) | 0.024 | -0.75 (-2.01, 0.52) | 0.249 |
| Seeing other services | 0.50 (-1.29, 2.29) | 0.584 | -0.33 (-1.99, 1.33) | 0.699 | -0.35 (-1.49, 0.78) | 0.542 | -0.35 (-1.50, 0.79) | 0.543 |
| **Participation Barriers** |  |  |  |  |  |  |  |  |
| *Family-related difficulties* |  |  |  |  |  |  |  |  |
| General family difficulties | -2.09 (-3.68, -0.51) | 0.010 | -1.16 (-3.44, 1.11) | 0.316 | -1.09 (-2.48, 0.30) | 0.123 | -0.84 (-2.20, 0.52) | 0.227 |
| Difficulties with own health | -2.14 (-3.61, -0.66) | 0.005 | -0.58 (-2.02, 0.86) | 0.432 | 0.46 (-0.53, 1.45) | 0.361 | -0.33 (-1.31, 0.66) | 0.515 |
| Child’s health/behaviour | -1.03 (-2.24, 0.18) | 0.096 | -0.58 (-1.56, 0.40) | 0.250 | -0.36 (-1.13, 0.41) | 0.358 | -0.92 (-1.73, -0.10) | 0.027 |
| Caring for other children | -0.13 (-1.61, 1.35) | 0.863 | -0.34 (-1.79, 1.11) | 0.642 | -0.48 (-1.54, 0.59) | 0.378 | 0.11 (-0.93, 1.14) | 0.841 |
| *Logistical difficulties* |  |  |  |  |  |  |  |  |
| Transport to and from group | -4.43 (-7.90, -0.96) | 0.012 | -1.14 (-3.53, 1.25) | 0.351 | -1.65 (-3.61, 0.32) | 0.101 | -2.73 (-4.65, -0.80) | 0.005 |
| Fitting in with child’s routine | -1.01 (-2.04, 0.02) | 0.055 | 0.52 (-0.41, 1.45) | 0.276 | -0.51 (-1.47, 0.45) | 0.299 | -0.58 (-1.56, 0.39) | 0.243 |
| Fitting in medical or other appointments | -3.04 (-4.56, -1.52) | <0.001 | -0.05 (-1.75, 1.65) | 0.957 | -0.59 (-1.67, 0.48) | 0.278 | -1.19 (-2.39, 0.00) | 0.050 |
| Work commitments | -3.10 (-5.43, -0.77) | 0.009 | -1.74 (-3.60, 0.13) | 0.068 | -1.98 (-3.33, -0.62) | 0.004 | -0.99 (-2.68, 0.69) | 0.248 |
| *Program-related difficulties* |  |  |  |  |  |  |  |  |
| Relating to other parents | -0.89 (-2.85, 1.06) | 0.372 | -0.21 (-1.77, 1.35) | 0.792 | -0.35 (-1.72, 1.01) | 0.610 | -0.42 (-1.71, 0.87) | 0.520 |
| Relating to staff | 0.00* |  | 0.04 (-4.69, 4.77) | 0.987 | -0.34 (-3.04, 2.36) | 0.807 | 0.09 (-3.38, 3.56) | 0.960 |
| Did not believe child was benefiting | 0.08 (-1.89, 2.05) | 0.933 | 0.65 (-1.40, 2.70) | 0.536 | -2.46 (-3.83, -1.09) | <0.001 | 0.45 (-1.39, 2.29) | 0.632 |
| **Program factors** |  |  |  |  |  |  |  |  |
| *Group Climate* |  |  |  |  |  |  |  |  |
| Group rapport | -1.07 (-4.50, 2.37) | 0.542 | 0.92 (-1.79, 3.63) | 0.507 | -0.32 (-2.43, 1.80) | 0.768 | 1.13 (-1.35, 3.62) | 0.371 |
| Time management | -0.72 (-4.69, 3.26) | 0.724 | -1.63 (-5.28, 2.02) | 0.382 | 0.42 (-1.98, 2.82) | 0.731 | 0.74 (-1.79, 3.28) | 0.566 |
| Session planning | 1.95 (-1.94, 5.84) | 0.326 | -0.76 (-3.92, 2.39) | 0.635 | -0.26 (-2.51, 1.98) | 0.817 | 0.30 (-2.04, 2.64) | 0.804 |
| Group cohesiveness | 0.95 (-1.43, 3.34) | 0.434 | 2.23 (0.70, 3.76) | 0.004 | 0.40 (-1.24, 2.03) | 0.635 | 1.05 (-0.81, 2.91) | 0.269 |
| Active participation | -0.34 (-2.69, 2.01) | 0.776 | 1.69 (0.10, 3.28) | 0.037 | 0.29 (-1.78, 2.36) | 0.782 | 1.23 (-0.69, 3.15) | 0.209 |
| Unanticipated events | -1.70 (-3.88, 0.49) | 0.128 | -1.78 (-3.42, -0.15) | 0.033 | -0.36 (-2.33, 1.61) | 0.717 | 0.65 (-1.91, 3.21) | 0.619 |
| Contact with family between sessions | -2.56 (-5.66, 0.53) | 0.105 | 3.68 (1.03, 6.33) | 0.006 | 1.18 (-1.74, 4.11) | 0.427 | 7.80 (5.22, 10.38) | <0.001 |
| *Facilitator Characteristics* |  |  |  |  |  |  |  |  |
| Age | -0.17 (-0.32, -0.02) | 0.024 | -0.16 (-0.27, -0.04) | 0.010 | 0.06 (-0.12, 0.24) | 0.539 | -0.05 (-0.24, 0.14) | 0.595 |
| Not university educated | 0.61 (-2.73, 3.95) | 0.720 | 1.32 (-1.71, 4.34) | 0.394 | -0.66 (-4.42, 3.10) | 0.731 | -0.39 (-3.19, 2.42) | 0.788 |
| Experience in early childcare | -0.08 (-0.27, 0.11) | 0.391 | -0.16 (-0.31, -0.00) | 0.045 | 0.05 (-0.11, 0.20) | 0.572 | 0.13 (-0.02, 0.28) | 0.090 |
| Experience with playgroups/parent groups | -1.36 (-5.93, 3.21) | 0.559 | -3.87 (-6.88, -0.87) | 0.012 | -0.01 (-2.98, 2.97) | 0.997 | -0.79 (-3.45, 1.87) | 0.560 |
| Knowledge/Skills about family support | -0.32 (-0.86, 0.22) | 0.246 | -0.57 (-1.13, -0.00) | 0.048 | 0.46 (0.15, 0.76) | 0.003 | 0.31 (-0.00, 0.63) | 0.052 |
| Training evaluation | -0.40 (-1.63, 0.83) | 0.528 | -0.57 (-1.57, 0.44) | 0.269 | 0.14 (-0.71, 0.98) | 0.751 | 0.01 (-0.73, 0.75) | 0.976 |

*Note:* Table shows regression coefficients with 95% confidence intervals (CI)

* 95% confidence interval and p-value could not be estimated
